# Identification of CD133-Positive Radioresistant Cells in Atypical Teratoid/ Rhabdoid Tumor

**DOI:** 10.1371/journal.pone.0002090

**Published:** 2008-05-07

**Authors:** Shih-Hwa Chiou, Chung-Lan Kao, Yi-Wei Chen, Chien-Shu Chien, Shih-Chieh Hung, Jeng-Fan Lo, Yann-Jang Chen, Hung-Hai Ku, Ming-Ta Hsu, Tai-Tong Wong

**Affiliations:** 1 Department of Medical Research and Education, Taipei Veterans General Hospital and National Yang-Ming University, Taipei, Taiwan; 2 Department of Physical Medicine and Rehabilitation, Taipei Veterans General Hospital and National Yang-Ming University, Taipei, Taiwan; 3 Cancer Center, Taipei Veterans General Hospital and National Yang-Ming University, Taipei, Taiwan; 4 The Neurological Institute, Section of Neuropediatric Surgery, Taipei Veterans General Hospital and National Yang-Ming University, Taipei, Taiwan; 5 Institute of Oral Biology, National Yang-Ming University, Taipei, Taiwan; 6 VYM Genomic Research Center, National Yang-Ming University, Taipei, Taiwan; 7 Institute of Anatomy and Cell Biology, National Yang-Ming University, Taipei, Taiwan; 8 Institute of Clinical Medicine, National Yang-Ming University, Taipei, Taiwan; Texas Tech University Health Sciences Center, United States of America

## Abstract

Atypical teratoid/rhabdoid tumor (AT/RT) is an extremely malignant neoplasm in the central nervous system (CNS) which occurs in infancy and childhood. Recent studies suggested that CD133 could be considered a marker for brain cancer stem-like cells (CSCs). However, the role of CD133 in AT/RT has never been investigated. Herein we report the isolation of CD133-positive cells (CD133^+^), found to have the potential to differentiate into three germ layer tissues, from tissues of nine AT/RT patients. The migration/invasion/malignancy and radioresistant capabilities of CD133^+^ were significantly augmented when compared to CD133^−^. The clinical data showed that the amount of CD133^+^ in AT/RTs correlated positively with the degree of resistance to radiation therapy. Using cDNA microarray analysis, the genotoxic–response profiles of CD133^+^ and CD133^−^ irradiated with 10 Gy ionizing radiation (IR) were analyzed 0.5, 2, 6, 12 and 24 h post-IR. We then validated these microarray data and showed increased phosphorylation after IR of p-ATM, p-RAD17, and p-CHX2 as well as increased expression of BCL-2 protein in CD133^+^ compared to CD133^−^. Furthermore, we found that CD133^+^ can effectively resist IR with cisplatin- and/or TRAIL-induced apoptosis. Immunohistochemical analysis confirmed the up-regulated expression of p-ATM and BCL-2 proteins in IR-treated CD133^+^ xenotransgrafts in SCID mice but not in IR-treated CD133^−^. Importantly, the effect of IR in CD133^+^ transplanted mice can be significantly improved by a combination of BCL-2 siRNA with debromohymenialdisine, an inhibitor of checkpoint kinases. In sum, this is the first report indicating that CD133^+^ AT/RT cells demonstrate the characteristics of CSCs. The IR-resistant and anti-apoptotic properties in CD133^+^ may reflect the clinical refractory malignancy of AT/RTs and thus the activated p-ATM pathway and BCL-2 expression in CD133^+^ could be possible targets to improve future treatment of deadly diseases like AT/RT.

## Introduction

Atypical teratoid/rhabdoid tumor (AT/RT), primary to the central nervous system (CNS), is a rare, aggressive, and highly malignant tumor which commonly occurs in patients under 3 years of age and is often fatal within 1 year after diagnosis [1]–[4]. Though usually described as extremely rare, AT/RT comprises up to 25% of primitive CNS tumors in infants [5]. In the past, the majority of AT/RTs were misclassified as primitive neuroectodermal tumors (PNET)/medulloblastoma (MB) at supratentorial sites because of the similarities in radiological and histological features of these two tumors [6], [7]. Many current treatment regimens for AT/RTs are derived from the strategies for PNET/MB, which are usually multimodal, and consist of radical surgery, chemotherapy, and radiotherapy. However, despite aggressive surgical and adjuvant radiochemotherapy, the outcome of AT/RT has been uniformly poor [8]–[10].

Because AT/RT can be difficult to distinguish from PNET/MB, the differential diagnosis of these tumors is very important [5], [9], [11], [12]. As the word *teratoid* indicates, AT/RTs show the multiple-lineage developmental characteristics of malignant teratomas of neuroectodermal, mesodermal, and endodermal lineages [6], [13], [14]. AT/RT expresses a wide range of immunohistochemical markers, such as vimentin, epithelial membrane antigen, cytokeratin, synaptophysin, glial fibrillary acidic protein, and smooth muscle actin [6], [13], [14]. Cytogenetic studies provide a useful tool in differential diagnosis of brain tumors. Previous studies have demonstrated 17p loss in 25–50% of medulloblastomas, but not in AT/RT [4]–[6]. Rorke et al further found that a subset of AT/RT contain the chromosomal abnormality, monosomy, or contain a deletion of chromosome 22 [15]. Recent studies reported that in a CNS rhaboid tumor an unbalanced 9;22 translocation leads to loss of 22q11 [16]. Subsequently, the hSNF5/INI1 gene on 22q11.2 was identified as a potential tumor suppressor gene responsible for the oncogenesis of AT/RT [17], [18].

Recently, CD133 (prominin-1: PROM1), a 5-transmembrane glycoprotein, was identified as an important marker representing a subset population of cancer stem-like cells (CSCs) in leukemia, retinoblastoma, colon cancer, prostate carcinoma, brain tumor, and hepatoma [19]–[23]. Bao et al. further demonstrated that the fraction of tumor cells expressing CD133, a marker for both neural stem cells and brain cancer stem cells, is enriched after radiation in gliomas [24]. Interestingly, these CD133-expressing CSCs play a critical role not only in the restoration of tumor cells and CSCs but also in the resistance to radiotherapy [24], [25]. In this study, using magnetic bead selection [19], [24], we successfully isolated CD133-positive cells from tissue samples of AT/RT patients. We found that CD133-positive AT/RT cells (CD133^+^) have a pluripotent differentiation ability and the capability of malignant cells to be highly resistant to ionizing radiation (IR). To further characterize the radioresistant properties and underlying pathogenesis of CD133^+^ cells in AT/RT, CD133^+^ and CD133-negative AT/RT cells (CD133^−^) were irradiated with 10 Gy IR and their genotoxic–response profiles analyzed by cDNA microarray. The microarray data indicated that CD133^+^ display greater checkpoint activation in response to DNA damage and higher antiapoptotic activity in radioresistance. Therefore, we further attempted to investigate the DNA repair and antiapoptotic activity of IR-treated CD133^+^, and to explore the possible mechanisms and therapeutic rationales associated with these radioresistant responses.

## Results

### Isolation and Characterization of CD133-positive cells from AT/RT tissues

**Figure 1:**
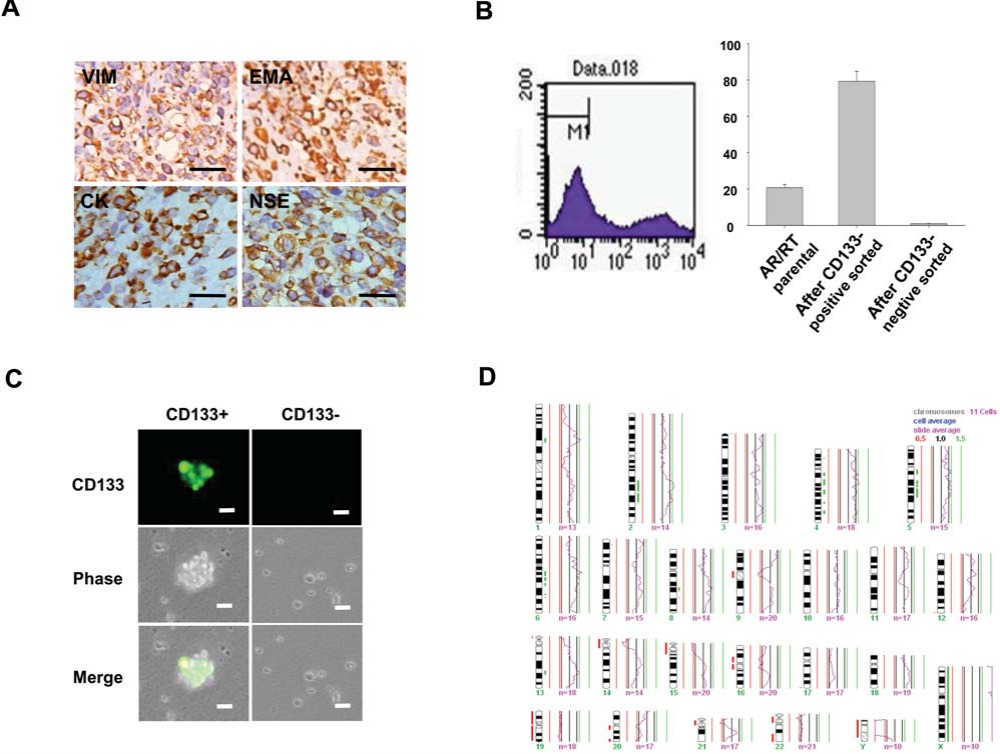
Isolation and Characterization of CD133^+^ from AT/RT tissues. (A) Imunohistochemical analysis of tumors from AT/RT patients for the AT/RT associated markers of vimentin (VIM), epithelial membrane antigen (EMA), cytokeratin (CK), and neuron-specific enolase (NSE). Using a magnetic bead method, CD133^+^ cells were isolated from 9 AT/RT patients, and identified by (B) Flow cytometry, and (C) Immunofluorescence (green fluorescence: positive for CD133 marker; bar: 20 µm). (D) The result of chromosomal analysis of CD133^+^ AT/RT cells analyzed by comparative genomic hybridisation (CGH). Data shown here are the mean±SD of three experiments.

A total of nine patients diagnosed with AT/RT are included in this study. Tumors from these nine patients were all positive for vimentin, epithelial membrane antigen, cytokeratin, neuron-specific enolase, glial fibrillary acidic protein, and synatophysin (Figure 1A and data not shown). Fourteen tumor samples from these nine patients were analysed by comparative genomic hybridization (CGH) and molecular analysis for *SMARCB1* (*hSNF5/INI1*). With the use of magnetic beads, CD133-positive AT/RT cells (CD133^+^) were isolated from the 14 tissue samples from the 9 AT/RT patients (Figure 1B; Table 1). Immunofluorescence confirmed that CD133 was highly expressed in sorted CD133^+^ (green fluorescence; Figure 1C), while there was no positive signal detected in CD133^−^ (Figure 1C). Furthermore, we analyzed these CD133^+^ for chromosomal abnormalities by CGH and molecular analysis for *SMARCB1*. The result showed that these CD133^+^ presented with the chromosomal abnormalities identified in a subset of AT/RT of monosomy, mutation, or deletion of chromosome 22, which are typical features of AT/RTs [17], [18]. (Figure 1D; Table 1)

**Table 1:** Case description and tumorigenic characteristics of CD133^+^ AT/RT.

					Number of cells injected (cell no.)	
Case	Age/Sex	Molecular Analysis*	Survival time	CD133^+^ (%)	CD133^+^	CD133^−^
1	0.7 / M	Positive	0.2 yr	36.4	10,000 (3/3)	10,000(0/3)
					3,000(3/3)	3,000(0/3)
					1,000(2/3)	1,000(0/3)
2	2.3 / F	Positive	0.3 yr	21.8 (69.5)**	10,000 (3/3)	10,000(0/3)
					3,000(3/3)	3,000(0/3)
					1,000(3/3)	1,000(0/3)
3	2.8 / M	Positive	4.4 yr	7.3 (29.6)**	10,000 (3/3)	10,000(0/3)
					3,000(3/3)	3,000(0/3)
					1,000(3/3)	1,000(0/3)
4	5.1 / M	Positive	1.7 yr	25.9 (43.1)**	10,000 (3/3)	10,000(0/3)
					3,000(3/3)	3,000(0/3)
					1,000(3/3)	1,000(0/3)
5	1.4 / M	Positive	8.7 yr	1.3	10,000 (2/3)	10,000(0/3)
					3,000(1/3)	3,000(0/3)
					1,000(0/3)	1,000(0/3)
6	3.3 / F	Positive	7.5 yr	1.6	10,000 (2/3)	10,000(0/3)
					3,000(1/3)	3,000(0/3)
					1,000(1/3)	1,000(0/3)
7	8.1 / F	Positive	4.7 yr	2.4 (37.5)**	10,000 (3/3)	10,000(0/3)
					3,000(3/3)	3,000(0/3)
					1,000(0/3)	1,000(0/3)
8	5.1 / M	Positive	1.7 yr	3.9 (48.5)**	10,000 (3/3)	10,000(0/3)
					3,000(3/3)	3,000(0/3)
					1,000(3/3)	1,000(0/3)
9	1.7 / M	Positive	2.5 yr	10.7	10,000 (3/3)	10,000(0/3)
					3,000(3/3)	3,000(0/3)
					1,000(2/3)	1,000(0/3)

* molecular analysis includes detection of deletion or mutation of 22q11.2 and *hSNF5/INI1* **gene and CGH findings**.

** the second surgery for tumor relapse. CD133^+^ and CD133^−^ cells were injected into the stratum of brains of SCID mice.

### Determination of cancer stem-like cell properties in CD133^+^ AT/RT cells

**Figure 2:**
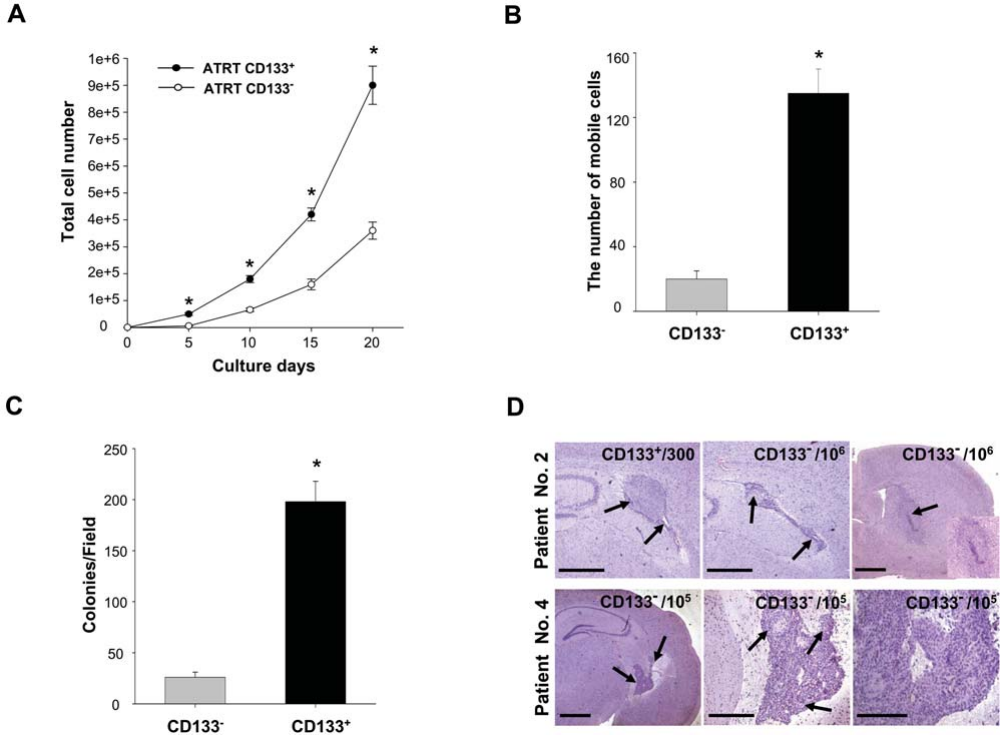
Cell growth rate, invasive ability, and tumor formation ability of CD133^+^/− AT/RT cells. The growth rate and tumorigenic ability of CD133^+^/− AT/RT cells were analyzed by using (A) MTT assay, (B) *In vitro* matrigel Transwell invasion assay, and (C) soft agar formation assay. *p<0.05 (D) The *in vivo* tumorigenic activity of CD133^+^/− AT/RT cells was assessed by injecting increasing numbers of cells into the brain stratum of SCID mice. Tumors develop with as few as 300 CD133^+^ AT/RT cells (Patient No. 2), whereas 10^5^ CD133^−^ (Patient No. 4) are needed to produce tumors. Bar: 150 m.

Having isolated the CD133^+^ cells from the AT/RT patients, we next determined their cancer stem-like cell properties. CD133^+^ had a higher proliferation rate than CD133^−^, as assessed by the MTT assay (p<0.05, Figure 2A), and were more invasive in *in vitro* matrigel Transwell invasion assays when compared to CD133^−^ (p<0.05; Figure 2B). When equal numbers of cells were plated in soft agar, CD133^+^ had a higher ability for tumor colony formation than CD133^−^ (p<0.05; Figure 2C). To determine the *in vivo* tumorigenic capacity of CD133^+^ and CD133^−^, increasing numbers (300, 10^3^, 3×10^3^, 10^4^) of cells were injected into the brain stratum of SCID mice. The results showed that 10^4^ CD133^−^ did not induce tumor formation but 3000 CD133^+^ from the nine patients (100%; Table 1) and 300 CD133^+^ from one of four patients (25%; Table 2) generated visible tumors in xenotransplanted mice 8 weeks after injection (Figure 2D; Patient No. 2). Moreover, one thousand CD133^+^ isolated from a transplanted brain tumor can further generate new (2^nd^) tumors (data not shown). To investigate further whether CD133^−^ AT/RT can generate tumors in transplant recipients, higher doses (10^5^ and 10^6^) of CD133^−^ isolated from 4 patients were injected into the brain striatum of SCID mice. The results showed that tumors can be detected in the brain lesions after 8 weeks when 10^5^ cells (25%; 1 of 4 patients; Patient No. 4) and 10^6^ cells (4 of 4 patients; 100%) of CD133^−^ AT/RT were implanted into SCID mice (Figure 2D; Table 2).

**Table 2:** Investigation of tumorigenicity frequency of CD133^+^ and CD133^−^ cells derived from four AT/RT patients in NOD/SCID xenotransplant assay.

	Cell Number for Injection				
Total of 4 Patients	300	10^3^	10^4^	10^5^	10^6^
CD133^+^	One of four	All positive	All positive	All positive)	All positive
	(25%)	(100%)	(100%)	(100%)	(100%)
CD133^−^	None	None	None	One of four	All positive
	(0%)	(0%)	(0%)	(25%)	(100%)

**Figure 3:**
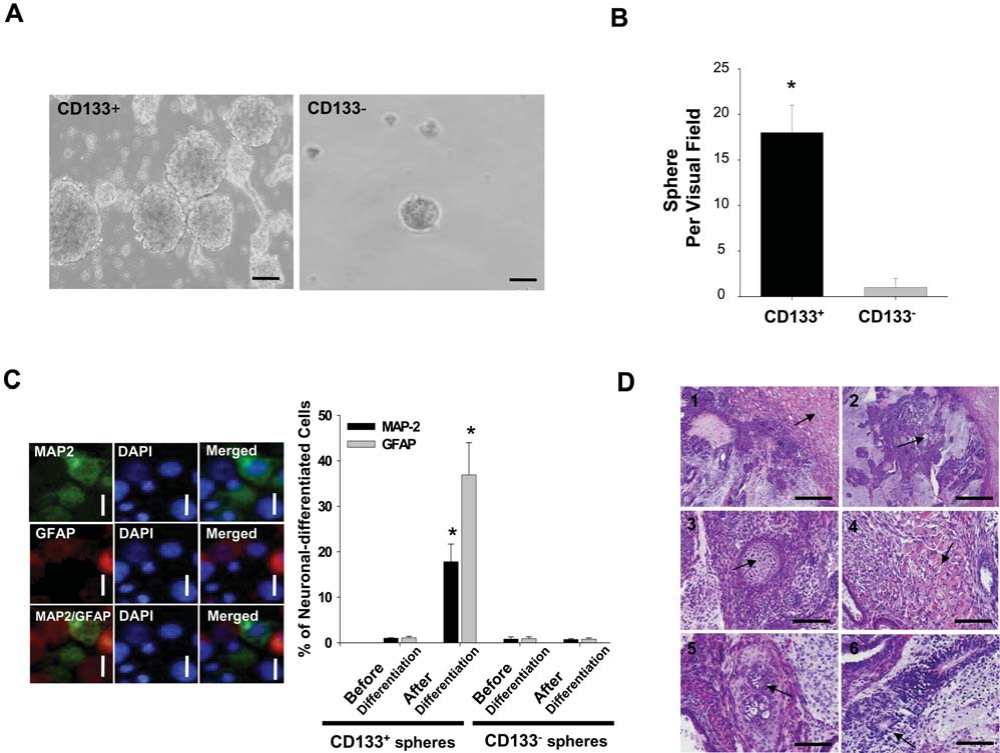
Multi-lineage differentiation capability of CD133^+^/− AT/RT cells. (A) and (B) sphere formation of CD133^+^/− AT/RT cells. CD133 cells were cultured in serum free medium with bFGF and EGF (20 ng/mL) for 4 weeks. (C) CD133^+^ AT/RT cells were cultured for 14 days on a poly-L-lysine coated plate in medium containing 2% FBS. The percentages of MAP-2-positive (MAP-2: neuron marker) and GFAP-positive cells (GFAP: glia marker) were detected in the differentiated CD133^+^ AT/RT cells. DAPI: staining for cell nuclei (blue fluorescent; bar: 20 µm).*p<0.05. (D) CD133^+^ AT/RT cells were injected into the subrenal space of a SCID mouse (n = 6). All the mice formed teratomas. (1) arrow: normal kidney tissue of the mouse. The three germ layers include (2) endothelium-like tissues (arrow), (3) epidermoid tissues (arrow), (4) muscle-like tissue (arrow), (5) cartilage-like tissues (arrow), and (6) neuroepithelium-like tissues (arrow); bar: 100 µm. Data shown here are the mean±SD of three experiments.

In addition, to investigate the stem-like cell properties of the CD133^+^ and CD133^−^ derived from AT/RT, the ability to form spheroid bodies and the multilineage differentiation ability were tested. Isolated CD133^+^ and CD133^−^ AT/RT cells were cultured in DF-12 serum-free medium with bFGF and EGF (20 ng/mL; Methods S1). After being in culture for 4 weeks, CD133^+^ aggregated and formed spheroid bodies (Figure 3A). The ability to form spheroid bodies (SB) in CD133^+^ AT/RT was significantly higher than that in CD133^−^ (p<0.05; Figure 3B). Immunofluorescence staining demonstrated that CD133^+^-SB can differentiate into MAP-2-positive (neuron marker) and GFAP-positive (astroglial marker) neuronal-like cells (Figure 3C). Furthermore, ten thousand cells from CD133^+^-SB were injected into the subrenal space of SCID mice. After 6 weeks, histological analysis indicated that teratoma-like tissues had formed at the injection site (Figure 3D). Importantly, we could demonstrate by hematoxylin and eosin staining development of the three germ layers including endothelium-like tissues (Figure 3D2), epidermoid tissues (Figure 3D3), muscle-like tissue (Figure 3D4), cartilage-like tissues (Figure 3D5), and neuroepithelium-like tissues (Figure 3D6). In contrast, CD133^−^ formed only a few spheres (CD133^−^-SB) after 4 weeks in serum-free medium with bFGF and EGF. CD133^−^-SB did not exhibit any multi-lineage differentiation *in vitro* (Figure 3C) and did not induce teratoma-like tissue *in vivo* (data not shown). Combining these results, our data indicated that CD133-positive cells isolated from AT/RT tissues present with the characteristics of cancer stem-like cells.

### Measurement of radiosensitivity in C133^+^/− AT/RT *in vitro* and *in vivo*

**Figure 4:**
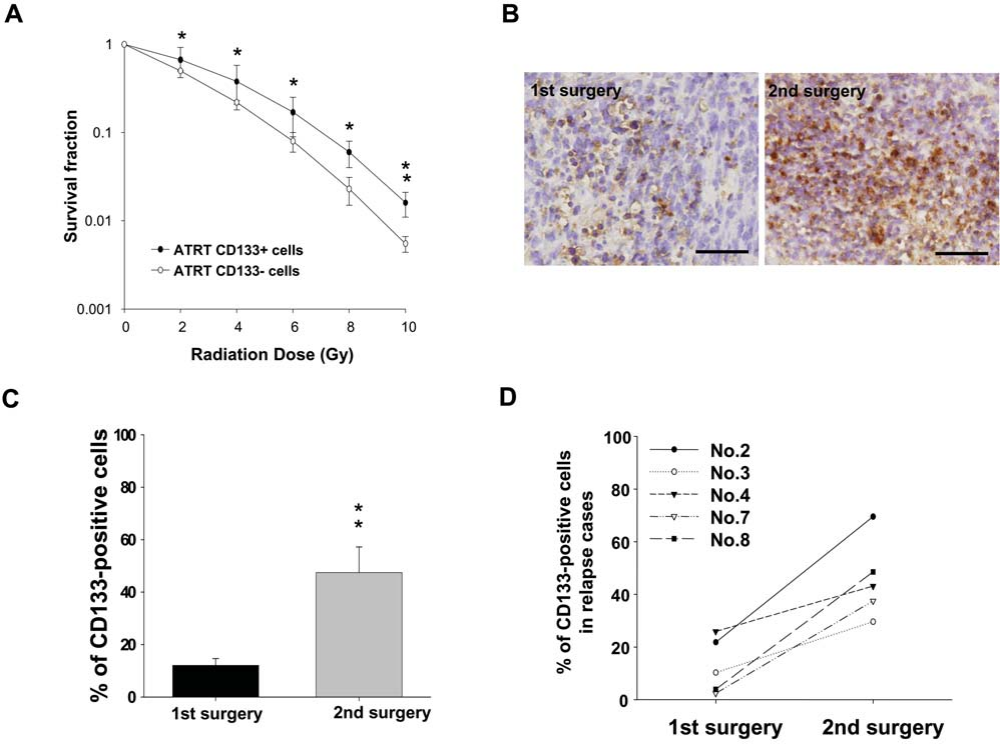
Evaluation of radiosensitivity in C133^+^/− AT/RT cells *in vitro* and *in vivo*. (A) The survival fraction of CD133^+^/− AT/RT cells after IR treatment. The IR dose: from 0 to 10 Gy. (B) Detection by immunohistochemistry of CD133^+^ AT/RT cells in the tissue samples of the same patient after the first surgery and the second surgery (tumor recurrence) (bar: 50 µm). (C) The percentage of CD133^+^ AT/RT cells (1^st^ surgery: 9 patients) was dramatically elevated in the tumor relapse samples (2^nd^ surgery: 5 patients). (D) Comparison of the tumor samples from the first and second surgeries in the five patients whose tumors relapsed. *p<0.05; **p<0.001. Data shown here are the mean±SD of three experiments.

To determine the effect of radiation on tumor growth rate, an ionizing radiation (IR) dose from 0 to 10 Gy was used to treat the two groups of cells. As shown in Figure 4A, the survival rate and number of CD133^+^ after IR treatment were significantly higher than those of CD133^−^ (p<0.05). In order to determine the effect of radiation on the *in vivo* proliferation abilities of CD133 cells, SCID mice were irradiated one week after CD133^+^ and CD133^−^ were injected into the brain stratum of SCID mice for analysis of *in vivo* tumorigenicity. The total volumes of CD133^+^ tumors in irradiated SCID mice were significantly higher than those of CD133^−^ AT/RT tumors in mice after IR treatment. Furthermore, there was no significant difference in the growth of CD133^+^ cells in irradiated SCID mice compared to non-irradiated mice (p>0.05; Data not shown).

Moreover, expression of CD133^+^ in the AT/RT patients during the course of treatment was investigated using flow cytometry and immunohistochemistry. As shown in Table 1, Patient No. 2, 3, 4, 7, and 8 received the complete course of radiotherapy combined with chemotherapy. However, the tumor relapsed and these five patients then underwent a second brain surgery. The percentage of CD133^+^ (Figure 4B&C) was dramatically elevated in the tumor relapse samples of these five patients as compared to the tumor samples from their first surgery (Figure 4D; Table 1). In addition, treatment efficacy and mean survival time of these 9 AT/RT patients significantly and negatively correlated with the levels of CD133^+^ cells in the patients' tissues (p<0.05; Table 1). Taken together, these data supported the claim that the amount of CD133^+^ cells in AT/RTs is strongly and positively correlated with the level of resistance to radio/chemotherapy as well as the occurrence of tumor relapse.

### The alteration of anti-apoptosis, cell cycle, and DNA repair gene clusters in CD133^+^/− AT/RT cells after ionizing radiation

**Figure 5:**
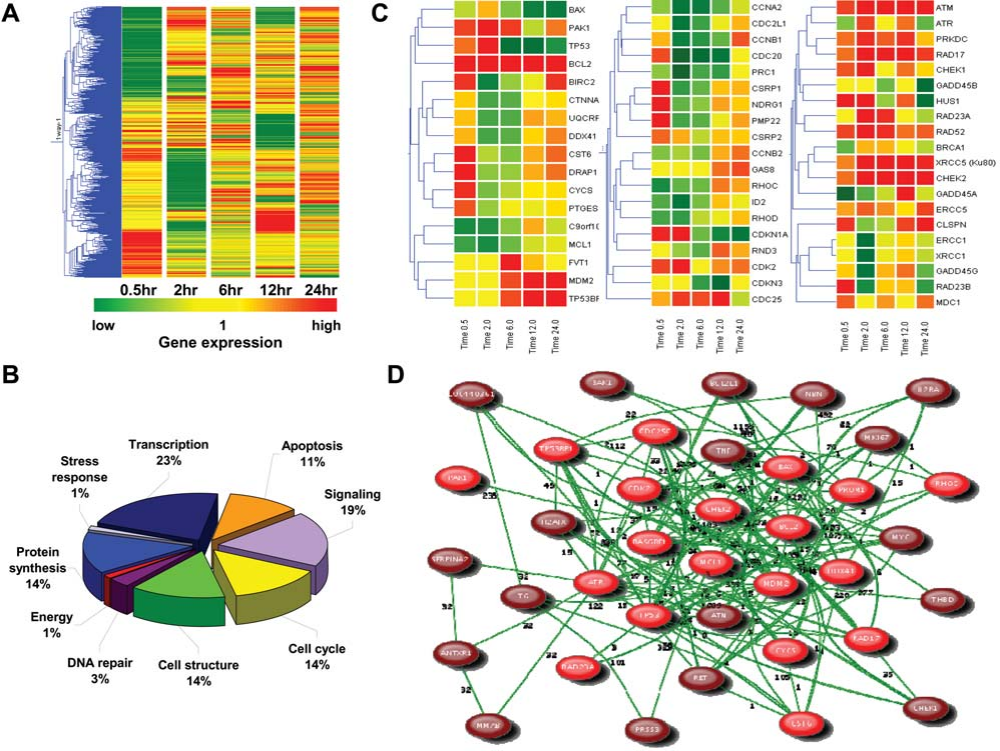
The alteration of anti-apoptosis, cell cycle, and DNA repair gene clusters in CD133^+^/− AT/RT cells after ionizing radiation (IR). (A) Gene tree for the experiments at 5 time points (0.5, 2, 6, 12 and 24 h post-IR) of the 1494 altered genes in IR-treated CD133^+^ AT/RT cells as compared to IR-treated CD133^−^ cells. The time dependent changes of 1494 altered genes are presented as a log scale of the expression values provided by GeneSpring GX software. (B) Molecular functions of the 327 significant genes (>two-fold up-regulation and <0.5-fold down-regulation) expressed in irradiated CD133^+^/− AT/RT cells. (C) There are three major gene groups were identified by GenSpring GX Gene Tree Clustering: anti-apoptosis and apoptosis, Cell Cycle, and DNA Repair. The correlations are indicated to the left of the corresponding nodes. Selected lots of functional genes are indicated to the right. The values given are the linear ratio of the average from three replicate experiments. (D) Thirty-seven literature-based network genes were involved and altered after IR. Of these 37 genes, 19 genes (Table S1) based on the results of our study were in rosy symbols and the co-expressed genes (sorted by PubGene) were in crimson. The expression presence of CD133 was directly correlated with BCL2, BCL2L1, BAX, TNF, MK167, IL2RA, TP53, and MDM2. Lines indicate co-citation in the literature in more than one article. The numbers indicate the number of Medline records containing the query term or one of its synonyms at least once. This tool allows us to explore in detail the literature associations between a set of genes, proteins or the combination of the two. The literature networks are based on the results of indexing using a Natural Language Processing (NLP) regimen of all MEDLINE records (title and abstract) for gene and protein names.

Microarray analysis showed that the expression of 1494 genes (Figure S1A–C) were significantly altered in IR-treated CD133^+^ as compared to IR-treated CD133^−^ at 0.5, 2, 6, 12 and 24 h post-IR when compiled with the hierarchical clustering method (Figure 5A). The time-dependent expression profiles of the 1494 genes were further analyzed by using GeneSpring software (Figure 5; Figure S1). A total of 327 genes (Figure 5B) differed significantly in their expression levels between IR-treated CD133^+^ and IR-treated CD133^−^ by more than two-fold (up-regulation) or less than 0.5-fold (down-regulation) (p<0.05). They can be divided into three major groups: (1) anti-apoptotic and apoptotic genes, (2) cell cycle-related genes, and (3) DNA repair-related genes, as visualized and analyzed by GenSpring GX Gene Tree Clustering (Figure 5C). Using real-time RT-PCR to confirm the microarray data, we showed that BCL-2 and BCL-XL were significantly up-regulated at 0.5, 2, 6, 12 and 24 h in irradiated CD133^+^ as compared to IR-treated CD133^−^ (Figure 2S). The expression level of CDKN1A (p21^Waf1/Cip1^) was up-regulated in CD133^+^ at 0.5 and 2 h post-IR, but showed significant down-regulation at 6, 12 and 24 h post-IR as compared to IR-treated CD133^−^ (Figure 2S). Moreover, the expression levels of TP53BP1 in CD133^+^ cells gradually increased from 2 h to 24 h post-IR as compared to IR-treated CD133^−^ cells (Figure 2S). In addition, the results of Western blot analysis were consistent with the gene expression profiles of BCL-2, BCL-XL, CDKN1A, and TP53BP1 in CD133^+^ cells after IR (Data not shown). Thus, the differential expression of genes associated with cell-cycle, growth, transcription signaling, anti-apoptosis, and apoptosis (Figure 5C and Figure 2S) could initiate signaling cascades leading to and/or preparing for the subsequent events of cell-cycle arrest, inhibition of proliferation and DNA repair, which could eventually lead to cell repair, regrowth, and mutagenesis in IR-treated CD133^+^ cells. In addition, we used a literature-based network analysis of all MEDLINE records (title and abstract) to group the target-linkage genes from our microarray data using a Natural Language Processing (NLP) regimen for gene and protein names. We identified thirty-seven literature-based network genes that were involved and altered after IR (Figure 5D and Table S1). Of these 37 genes, 19 genes (Table S1) are in rosy symbols and the co-expressed genes (sorted by PubGene) are in crimson. The results of this literature linkage analysis support the microarray data which suggest that CD133 expression is involved in the activation of anti-apoptosis, cell cycle, and DNA repair related gene clusters.

### Increased phosphorylation of ATM-related proteins and expression of BCL-2 protein in CD133^+^ AT/RT cells *in vitro* and *in vivo* after IR

**Figure 6:**
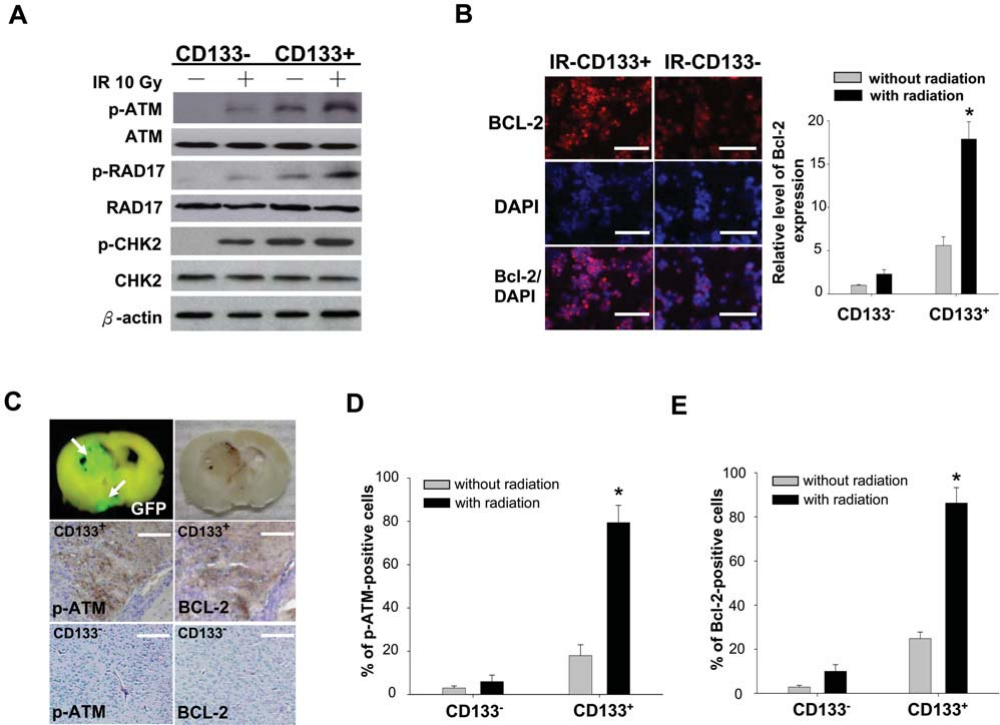
Detection of phosphorylated ATM-related proteins and of BCL-2 protein in CD133^+^/− AT/RT after IR. (A) Using Western blotting analysis, increased levels of phosphorylation of p-ATM, p-RAD17, and p-CHK2 were detected in IR-treated CD133^+^ AT/RT cells compared to IR-treated CD133^−^ AT/RT cells. (B) Immunofluorescent staining of BCL-2 protein in CD133^+^/− AT/RT cells before and after IR. Bar: 100 µm. (C) To further study protein expression in CD133^+^/− AT/RT xenotransplanted grafts in SCID mice with or without IR, tumor growth in the brain of SCID mice was evaluated by *in vivo* GFP imaging and immunohistochemistry (IHC). The expression of (D) p-ATM protein and (E) BCL-2 protein in the brain lesions of CD133^+^/− AT/RT -injected mice were detected by IHC. Bar: 150 µm. *p<0.05. Data shown are the mean±SD of three experiments.

To further validate DNA damage checkpoint responses in CD133^+^ radioresistant AT/RT cells, we compared early ATM-related DNA damage responses in CD133^+^ and CD133^−^ subpopulations of AT/RT cells. Consistent with our microarray findings, the activating phosphorylation of the checkpoint proteins, p-ATM, p-RAD17, and p-CHK2, was significantly higher in IR-treated CD133^+^ than in IR-treated CD133^−^ (Figure 6A), indicating that CD133^+^ AT/RT cells display greater checkpoint activation in response to DNA damage. Immunofluorescence analysis confirmed that the expression of BCL-2 in IR-treated CD133^+^ was significantly higher than that in IR-treated CD133^−^ (p<0.05; Figure 6B). Interestingly, it was also noted that higher endogenous levels of BCL-2 protein and of phosphorylated ATM, RAD17 and CHK2 were detected in CD133^+^ without any treatment (Figures 6A & 6B). Moreover, we investigated whether the up-regulated expression of BCL-2 can increase the IR-resistant and anti-apoptotic activities in IR-treated CD133. Irradiated CD133 cells were treated with cisplatin (1 µg/ml) or TRAIL (100 ng/ml) alone, or in combination. As shown in Figure S3A, the cell survival rate in IR-treated CD133^+^ was not significantly decreased by the IR treatment combined with cisplatin, with or without TRAIL treatment. In contrast, cell survival in CD133^−^ declined significantly after treatment with cisplatin alone and in combination with TRAIL (Figure S3A). IR with cisplatin or TRAIL alone, or in combination, increased caspase 3 activity in CD133^−^, however, there was no significant difference in caspase 3 activity in similarly treated CD133^+^ (Figure S3B).

In order to further confirm whether the *in vivo* expression of phosphorylated ATM and BCL-2 protein in transplanted mice was also influenced by IR treatment, SCID mice transplanted with CD133^+^ or CD133^−^ received irradiation with or without cisplastin. Immunohistochemical analysis (Figure 6C) demonstrated that both p-ATM (Figure 6D) and BCL-2 protein (Figure 6E) were up-regulated more in IR-treated CD133^+^ than in IR-treated CD133^−^. Secondly, using an *in vivo* GFP imaging system to visualize the tumor [26], the tumor volume of CD133^+^ cannot be effectively diminished by IR treatment alone, cisplatin alone, or a combination of IR/cisplatin as compared to the same treatment of CD133^−^ (Figure S3C). Finally, Kaplan-Meier survival analysis indicated that the mean survival rate of mice with IR/cisplatin-treated CD133^+^ was significantly lower than those with CD133^−^ or IR/cisplatin-treated CD133^−^ (Data not shown).

### Enhanced radiosensitivity of CD133^+^ AT/RT cells after combined treatment with a checkpoint kinases inhibitor and BCL-2 siRNA

**Figure 7:**
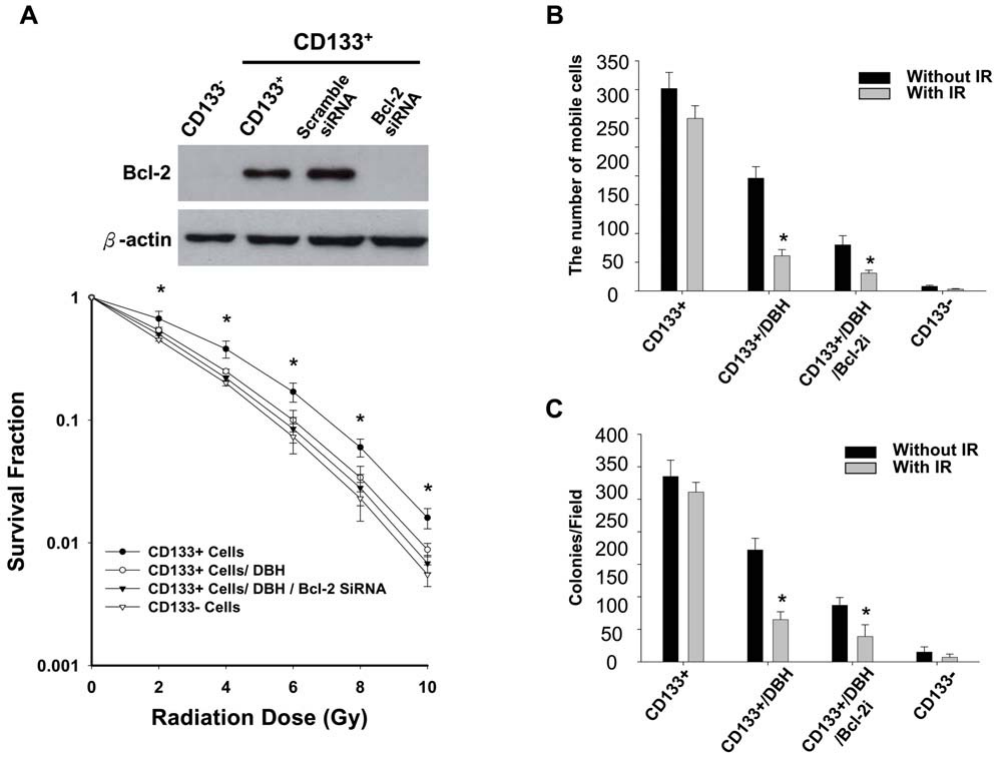
Enhanced radiosensitivity of CD133^+^ AT/RT cells after combined treatment with a checkpoint kinases inhibitor and BCL-2 siRNA. (A) Western blott data showed that the protein levels of BCL-2 in CD133^+^ AT/RT were significantly upregulated compared with those of CD133^−^. Treatment of BCL-2 siRNA can efficiently block the protein expression of BCL-2 in CD133^+^ (upper part). The effect of IR on CD133^+^ AT/RT cells was significantly improved by DBH alone or DBH in comination with BCL-2 siRNA (lower part). The tumorigenic properties of (B) migration/invasion and (C) tumor colony formation were significantly inhibited in CD133− AT/RT cells treated by DBH alone or DBH combined with BCL-2 siRNA as compared to CD133^+^ and IR-treated only CD133^+^ groups. *p<0.05. Data shown are the mean±SD of three experiments.

To further investigate the role of the increased phosphorylation of ATM-pathway proteins and expression of BCL-2 protein in CD133^+^ AT/RT, we treated the cells with debromohymenialdisine (DBH; an inhibitor of checkpoint kinases, 3 mM; Calbiochem, USA) alone or in combination with silencing of the BCL-2 gene [27] with small interfering RNA (siRNA) with a lentiviral vector (Figure 7A; Methods S1). The result showed that the effect of IR on CD133^+^ can be significantly improved by DBH alone or DBH in combination with BCL-2 siRNA (Figure 7A). Compared with IR-treated only CD133^+^, the tumorigenic properties of migration/invasion (Figure 7B) and tumor colony formation (Figure 7C) were significantly inhibited in CD133^+^ treated with DBH alone or DBH combined with BCL-2 siRNA. These data indicated that the radioresistance of CD133^+^ to IR is partially due to preferential activation of checkpoint kinases and Bcl-2 proteins.

**Figure 8:**
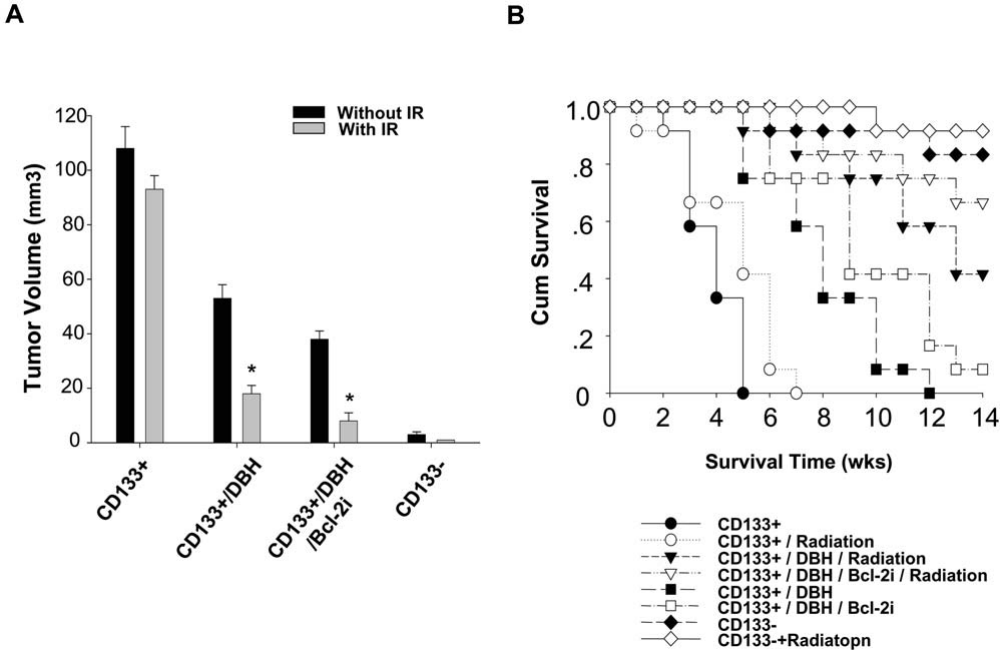
Significantly improved tumor growth and prolonged survival rate in CD133^+^ AT/RT cells transplanted SCID mice by the combined treatment of a checkpoint kinases inhibitor and BCL-2 siRNA. (A) The tumorigenicity analysis showed that the number of tumor foci and tumor volumes in tumor-bearing SCID mice treated by DBH+IR alone or DBH+IR combined with BCL-2 siRNA were significantly lower than those of CD133^+^ or only IR-treated CD133^+^. Moreover, the effectiveness of radiotherapy for CD133^+^ cells can be also improved by the treatment with DBH in combination with BCL-2 siRNA (*p<0.05: CD133^+^/DBH Vs. CD133^+^/DBH/IR; CD133^+^/DBH/BCL-2 siRNA Vs. CD133^+^/DBH/BCL-2 siRNA/IR). (B) Kaplan-Meier survival analysis further indicated that the mean survival rates in CD133^+^ treated by DBH+IR alone or DBH+IR combined with BCL-2 siRNA group can be significantly prolonged compared to CD133^+^ and only IR-treated CD133^+^ groups. Data shown are the mean±SD of three experiments.

In order to further confirm the proliferative and IR-resistant abilities of CD133^+^ AT/RT *in vivo*, the eight groups - CD133^+^, CD133^−^, only IR-treated CD133^+^, only IR-treated CD133^−^, DBH-treated CD133^+^, DBH/IR-treated CD133^+^, DBH/BCL-2 siRNA-treated CD133^+^, and DBH/BCL-2 siRNA/IR-treated CD133^+^ were individually injected into the tail vein of SCID mice for xenotransplanted tumorigenicity analysis. The tumor foci and volumes of CD133^+^ treated by DBH+IR alone or DBH+IR combined with BCL-2 siRNA were significantly decreased compared to those of CD133^+^ and only IR-treated CD133^+^ (p<0.01; Fig. 8A). Importantly, Kaplan-Meier survival analysis further indicated that the mean survival rates in CD133^+^ treated by DBH+IR alone or DBH+IR combined with BCL-2 siRNA can be significantly prolonged compared to CD133^+^ and only IR-treated CD133^+^ (p<0.01; Fig. 8B). Moreover, *in vivo* study also confirmed that the effectiveness of chemotherapy for CD133^+^ cells can be also improved by the treatment with DBH in combination with BCL-2 siRNA (data not shown). These data provide evidence that targeting the p-ATM pathway and BCL-2 in CD133^+^ cells will be vital for improving the treatment of deadly diseases like AT/RT.

## Discussion

Radiotherapy and chemotherapy play significant and crucial roles in prolonging cancer patient survival, and a recent study showed that radiotherapy is promising and might be more efficacious than chemotherapy for AT/RT patients [8]–[10]. However, there is still a high failure rate and low median survival in AT/RTs undergoing radiotherapy when compared to patients with other pediatric CNS tumors, like medulloblastoma [9], [10]. In this study, we isolated CD133^+^ cells from nine AT/RT patients (Figure 1) and found that the percentage of CD133^+^ strongly and negatively correlated with the clinical outcome (mean survival time and treatment efficacy) of AT/RT patients (Table 1). In five AT/RT patients whose tumors relapsed after radiochemotherapy, the percentage of CD133^+^ in the relapsed tumor was significantly higher than in the original tumor (Figure 4; Table 1). We also found that CD133^+^ isolated from the nine AT/RT patients expressed severe deletion of chromosome 22q11 (Figure 1; Table 1), and these cells further demonstrated a self-renewal capability, rapid growth rate, and multipotential to differentiate into three germ layers (Figures 2 & 3). The migration, invasion, malignancy, and radioresistant capabilities, shown by *in vitro* and *in vivo* assays, of CD133^+^ cells were also significantly higher than those of the parental CD133^−^ cells (Figures 2 & 4). CD133^+^ AT/RT cells preferentially activate DNA damage checkpoint (p-ATM, p-RAD17, and p-CHK2) and antiapoptotic (BCL-2 and BCL-XL) genes in response to IR (Figure 5; Figure S1 & Figure S2). Furthermore, they repair radiation-induced DNA damage and anti-apoptotic activity more effectively than CD133^−^ AT/RT cells. The results of *in vivo* GFP imaging and immunohistochemical analyses confirmed that the up-regulated expression of p-ATM and BCL-2 proteins positively correlated with the failure rates of radiochemotherapy, and negatively correlated with the mean survival times of IR-treated CD133^+^ xenotransplanted mice as compared to the CD133^−^ transplanted mice (Figure 6; Figure S3). Importantly, the radiation- and/or chemodrug- resistance of CD133^+^ AT/RT *in vitro* and *in vivo* could be significantly reversed by treatment with DBH (a specific inhibitor of CHK1 and CHK2) combined with BCL-2 siRNA (Figure 7). In addition, Kaplan-Meier survival analysis further indicated that the mean survival rate of mice with CD133^+^ under radiation treatment could be significantly improved when combined with DBH alone or DBH together with BCL-2 siRNA (Figure 8). To our knowledge, this is the first study to report the presence of CD133^+^ in AT/RT, and to show that the activated p-ATM pathway and BCL-2-related antiapoptotic activity in CD133^+^ and IR-CD133^+^ render the CD133^+^ subpopulation capable of causing radio/chemoresistance in malignant tumors.

CD133 has been considered an important marker of a subset of CSCs in brain tumors [19]–[22]. A recent report suggested that expression of CD133 antigen in glioma could serve as a prognostic indicator for tumor regrowth, malignant progression, and patient survival [28]. In our study investigating the role of CD133^+^ in the tumorigenicity of AT/RT, we found that as few as 300 CD133^+^ AT/RT cells could induce tumor formation in xenotransplanted mice (Table 2; Figure 2D). Our data indicated that CD133^+^ contain the self-renewing and repopulation capabilities *in vitro* and *in vivo* that CD133^−^ AT/RTs lack unless a high cell number (10^5^ to 10^6^) was used as an inoculum in SCID mice (Figure 2, Table 2). Similar to our finding, Beier et al. has suggested that CD133^−^ glioblastoma cells possess a small subpopulation of cancer stem cells which have the ability to drive tumor growth *in vivo* [29]. Thus, whether CD133 is indeed the novel surface marker which allows the separation of cancer stem cells or tumor-initiating cells from brain tumors needs further investigation.

Several genes known to be involved in DNA damage repair (double strand-break rejoining), such as the ATM, GADD45, Ku80 (XRCC5), and ERCC5, showed increased expression in CD133^+^ AT/RT cells post IR treatment (Figure 5). ATM, a protein defective in the heritable disorder, ataxia telangiectasia, is a central signaling kinase in the response to double strand breaks and is involved in the regulation of cell cycle checkpoints [30]–[32]. Recently, Bao et al. demonstrated that IR-treated CD133^+^ glioma cells can activate ATM-related DNA damage checkpoint responses. They suggested that the radioresistance of CD133^+^ cells is triggered by preferential checkpoint activation [24]. In agreement with their findings, our results revealed activated and phosphorylated ATM and its downstream effectors (Figures 5, 6; Figure S2), CHEK1, CHEK2, MDC1, RAD17, RAD23A, RAD52, CDK2, CDC25, and TP53BP1, which were also up-regulated in CD133^+^ AT/RT cells in response to IR. Importantly, Mirzayans et al. reported that ATM-deficient cells exhibited marked radiosensitivity and p53-deficient cells had varying degrees of radioresistance compared with normal fibroblasts [33]. The expression of tumor protein p53 (TP53) in CD133^+^ AT/RT cells was first up-regulated 2 hours after IR but was then significantly inhibited 4 hours after IR (Figure 5; Figure S2). Cyclin-dependent kinase inhibitor 1A (p21, Cip1), the downstream protein of TP53 [34]–[36], presented a similar pattern in irradiated CD133^+^ AT/RT cells (Figure 5; Figure S2). Interestingly, we found that the levels of MDM2, a nuclear phosphoprotein which binds to TP53 [37], were significantly up-regulated in CD133^+^ AT/RT cells 4 hours after IR (Figure 5; Figure S2). Indeed, over-expression of MDM2 can result in excessive inactivation of TP53, diminishing its tumor suppressor function [37], [38].

Expression of the apoptosis gene BAX was slightly increased in CD133^+^ AT/RT cells 2 hours after IR and significantly suppressed 4 hours after IR (Figure 5; Figure S2). In contrast, expression of the anti-apoptotic genes BCL-2 and BCL-XL was significantly and rapidly (2 h) up-regulated in CD133^+^ AT/RT cells and remained elevated 24 hours post IR (Figure 5; Figure S2). Over-expression of BCL-2 in malignant laryngeal cancer is considered a contributing factor to radiotherapy failure [39]. The induction of a radioadaptive response in human lymphoblastoid cells can be abrogated by either loss of TP53 or BCL-2 over-expression [40]. Recently, increased phosphorylation of ATM (ATMpSer1981) and CHK2 (CHK2pTHr68) was specifically found in the precursors of prostatic carcinoma [41]. Consistent with these findings, over-expressed p-ATM and BCL-2 proteins positively and significantly correlated with radioresistant responses and high mortality rates *in vitro* and *in vivo* in our IR-treated CD133^+^ (Figure 6; Figure S3). According to the functional-linkage analysis of literature-based network from the microarray data, CD133 (PROM1) plays a role in maintaining a relationship between BCL-2, BAX, CHEK2, MDM2, ATM, TP53, ATR, CDK2, CDC25C, TP53BP1 (Figure 5D and Table S1). Thus, our results imply that CD133^+^ AT/RT cells represent the cellular population which confers AT/RT radioresistance, and could be the source of tumor recurrence after radiation.

In conclusion, our data indicate that CD133^+^ AT/RTs present both the characteristics of stem cells and malignant tumors. The radio/chemoresistant and anti-apoptotic properties in CD133^+^ AT/RT cells may reflect the clinical refractory malignancy of AT/RTs as well as cancer stem-like cells. This CSCs property in AT/RT and other tumors should be considered in future translational oncology as instrumental to ultimately improving anti-cancer therapies. In addition, the activated p-ATM-related DNA repair pathway and anti-apoptotic genes in the CD133^+^ subset could be warranted as possible targets to improve the therapy for the treatment of advanced malignant brain tumors.

## Materials and Methods

### Isolation of CD133^+^ cell subsets from AT/RT tissues

This research followed the tenets of the Declaration of Helsinki and all samples were obtained after patients had given their informed consent. The dissociated cells from the samples of brain tumors from AT/RT patients were labeled with 1 mL CD133/l micromagnetic beads per 1 million cells using the CD133 cell isolation kit (MACS, Miltenyi Biotec). CD133^+^ cells were cultured in serum-free DMEM/F12 (GIBCO) medium, supplemented with N2 supplement (R&D), 10 ng/mL human recombinant bFGF (R&D) and 10 ng/mL EGF [42]. The Gamma Radiation was delivered by Theratronic cobalt unit T-1000 (Theratronic Internation, Inc., Ottawa, Canada) at a dose rate of 1.1 Gy/min (SSD = 57.5 cm). For the evaluation of cell proliferation rate, cells were seeded on 24-well plates at a density of 2×10^4^ cells/well in medium, followed by the methyl thiazol tetrazolium assay (MTT assay; Sigma-Aldrich Co.). The amount of MTT formazan product was determined using a microplate reader and an absorbance of 560 nm (SpectraMax 250, Molecular Devices, Sunnyvale, CA, USA).

### Comparative genomic hybridisation (CGH)

The CGH procedure was described in a previous publication [43], namely, metaphase spreads from the patient lymphocytes were prepared using standard protocols. Nick-translated, spectrum red-labeled tumor DNA and spectrum green-labeled normal DNA were coprecipitated with excess unlabeled human *Cot*-1 DNA (GibcoBRL), denatured, and hybridized to the normal metaphase slide preparations. Ten to twelve images were captured and analysed using a Cytovision workstation. The threshold indicated that gain and loss were set at 1.2 and 0.8, respectively.

### Microarray analysis and real-time RT-PCR

Total RNA was extracted from CD133^+^ and CD133^−^ AT/RT cells using Trizol reagent (Life Technologies, Bethesda, MD, USA) and the Qiagen RNAeasy (Qiagen, Valencia, CA, USA) column for purification. Total RNA was reverse-transcribed with Superscript II Rnase H-reverse transcriptase (Gibco BRL) to generate Cy3-and Cy5-labeled (Amersham Biosciences Co., Piscataway, NJ, USA) cDNA probes for control and treated samples, respectively. The labeled probes were hybridized to a cDNA microarray containing 10,000 gene clone immobilized cDNA fragments [43]. Fluorescence intensities of Cy3 and Cy5 targets were measured and scanned separately using GenePix 4000B Array Scanner (Axon Instruments, Burlingame, CA, USA). Data analysis was performed using GenePix Pro 3.0.5.56 (Axon Instruments, USA) and GeneSpring GX 7.3.1 software (Agilent, Palo Alto, CA). The method of real-time RT-PCR was performed as described [44]. Briefly, total RNA (1 µg) of each sample was reversely transcribed in 20 µL using 0.5 µg of oligo dT and 200 U Superscript II RT (Invitrogen, Carlsbad, CA). The primer sequences used in real-time RT-PCR are shown in Table S2. The amplification was carried out in a total volume of 20 µl containing 0.5 µM of each primer, 4 mM MgCl_2_, 2 µl LightCycler™–FastStart DNA Master SYBR green I (Roche Molecular Systems, Alameda, CA) and 2 µl of 1∶10 diluted cDNA. PCR reactions were prepared in duplicate and heated to 95°C for 10 minutes followed by 40 cycles of denaturation at 95°C for 10 seconds, annealing at 55°C for 5 seconds, and extension at 72°C for 20 seconds. Standard curves (cycle threshold values versus template concentration) were prepared for each target gene and for the endogenous reference (GAPDH) in each sample. The quantification of the unknown samples was performed by the LightCycler Relative Quantification Software version 3.3 (Roche Molecular Systems, Alameda, CA).

### In vitro cell invasion analysis and soft agar assay

The 24-well plate Transwell® system with a polycarbonate filter membrane of 8-µm pore size (Corning, United Kingdom) was used. The cell suspensions were seeded to the upper compartment of the Transwell chamber at the cell density of 1×10^5^ in 100 µl serum free medium. After 24 hours, the medium was removed and the filter membrane was fixed with 4% formalin for 1 hour. The opposite surface of the filter membrane which faced the lower chamber was stained with Hoechst 33342 for 3 mins and the migrated cells were then visualized under an inverted microscope. The protocol of soft agar assay was performed as follows. Each well (35 mm) of a six-well culture dish was coated with 2 ml bottom agar mixture (DMEM, 10% (v/v) FCS, 0.6% (w/v) agar). After the bottom layer had solidified, 2 ml top agar-medium mixture (DMEM, 10% (v/v) FCS, 0.3% (w/v) agar) containing 2×10^4^ cells was added, and the dishes were incubated at 37°C for 4 weeks. The plates were stained with 0.5 ml of 0.005% Crystal Violet for 1 hour, then the number of colonies was counted by a dissecting microscope [44].

### Immunofluorescence staining and immunohistochemistry

The protocol followed is the one described in the previous study [45]. Briefly, an avidin-biotin complex method was used for the immunofluorescence staining in the differentiated spheroid and neuronal-like cells. Each slide was treated with antibodies for CD133 (MACS, Miltenyi Biotec), GFAP (Chemicon), and MAP2 (Chemicon), phospho-ATM (Ser-1981; Upstate, Lake Placid, NY) and BCL-2 (Chemicon). Immunoreactive signals were detected with a mixture of biotinylated rabbit antimouse IgG and Fluoesave (Calbiochem, La Jolla).

### In vivo analysis of tumor growth and metastasis

All procedures involving animals were in accordance with the institutional animal welfare guideline of Taipei Veterans General Hospital. 2×10^4^ cells CD133^+^ and CD133^−^ AT/RT cells were injected into the brain stratum of SCID mice (BALB/c strain) each aged 8 weeks. *In vivo* GFP imaging was visualized and measured by an illuminating device (LT-9500 Illumatool TLS equipped with excitation illuminating source (470 nm) and filter plate (515 nm)). The tumor size was measured by a caliper and the volume was calculated according to the formula: (Length×Width^2^)/2. The integrated optical density of green fluorescence intensity was captured and then analyzed by Image Pro-plus software [26].

### Statistical analysis

The results are reported as mean±SD. Statistical analysis was performed using Student's-t test or the one-way or two-way ANOVA test followed by Turkey's test, as appropriate. A p<0.05 was considered to be statistically significant.

## Supporting Information

Figure S1 The time-dependent expression profiles of the 1494 genes were further analyzed by GeneSpring software. (A–C) Molecular functions of the 1494 genes expressed in irradiated CD133+/− AT/RT cells.
(0.23 MB TIF)

Figure S2 Detection of mRNA expression levels in CD133+/− AT/RT cells before and after IR. The expression levels of BCL-2, BCL-XL, BAX, CDKN1A, TP53BP1, and MDM2 in CD133+ AT/RT cells as compared to CD133− AT/RT cells by real-time RT-PCR at 0.5, 2, 6, 12 and 24 h post-IR. Data shown here are the mean +/− SD of three experiments.
(2.61 MB DOC)

Figure S3 Investigation of the sensitivity to radiochemotherapy and the antiapoptotic ability of CD133+/− AT/RT cells. (A) The cell survival rate in IR-treated CD133+/− AT/RT cells before and after treatment with cisplatin (1 ug/ml), TRAIL (100 ng/ml) or a combination of cisplatin and TRAIL. (B) Caspase 3 activity was detected by ELISA in IR-treated CD133+/− AT/RT cells before and after treatment with cisplatin, TRAIL or a combination of cisplatin and TRAIL. *p<0.001. Data shown are the mean +/− SD of three experiments. (C) The tumor volume in CD133+/− AT/RT transplanted SCID mice treated with IR alone, cisplatin alone, and IR+cisplatin, as measured by GFP imaging and histology. Data shown are the mean +/− SD of three experiments.
(2.79 MB DOC)

Methods S1 (0.10 MB DOC)

Table S1 (0.06 MB DOC)

Table S2 (0.04 MB DOC)
