# Supplementary figures and images for: Identification of CD133-Positive Radioresistant Cells in Atypical Teratoid/ Rhabdoid Tumor
Source: PLoS One. 2008 May 7;3(5):e2090. doi: 10.1371/journal.pone.0002090 (PMC2396792; doi:10.1371/journal.pone.0002090)

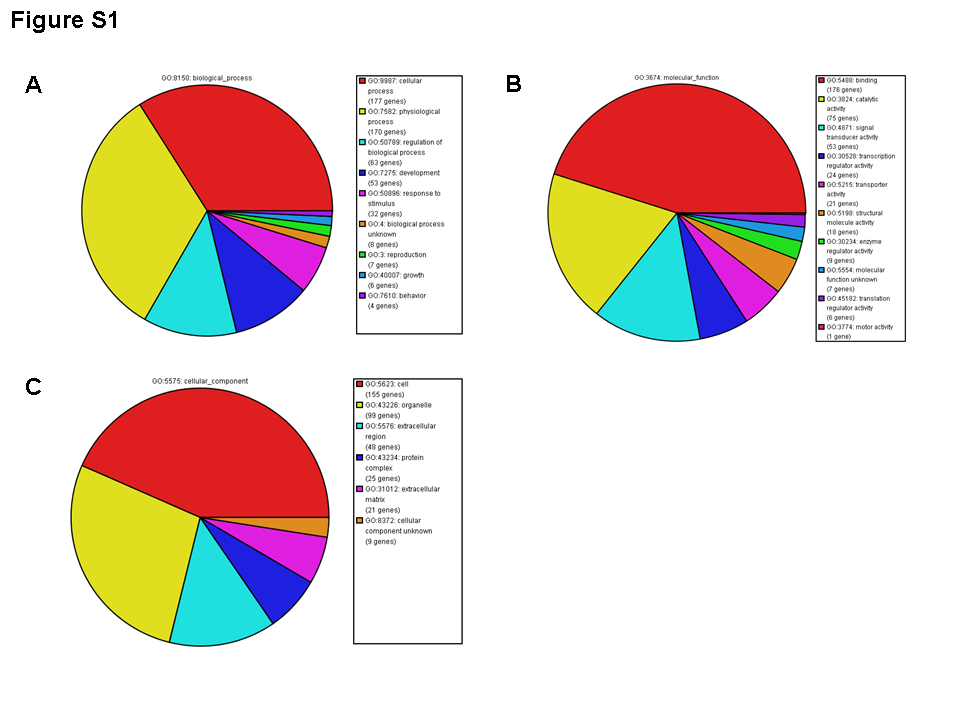

Supplement: Figure S1 — The time-dependent expression profiles of the 1494 genes were further analyzed by GeneSpring software. (A–C) Molecular functions of the 1494 genes expressed in irradiated CD133+/− AT/RT cells. (0.23 MB TIF) [file pone.0002090.s001.tif]
