# Supplementary material for: Identification of CD133-Positive Radioresistant Cells in Atypical Teratoid/ Rhabdoid Tumor
Source: PLoS One. 2008 May 7;3(5):e2090. doi: 10.1371/journal.pone.0002090 (PMC2396792; doi:10.1371/journal.pone.0002090)
Supplement: Methods S1 — (0.10 MB DOC) [file pone.0002090.s004.doc]

**Methods S1**

**The isolation and culture protocol of CD133-positive/cancer stem-like AT/AT cells.**

Tissues from the brain lesions of AT/RT patients were dissociated and incubated in Hank's balanced salt solution (HBSS) containing collagenase (78 units/ml) and hyaluronidase (38 units/ml) for 10 mins at 37℃. The tissues were then mechanically dissected and placed in a trypsin solution (1.33 mg/ml) at 37°C for another 10 mins. Dissociated cells were then centrifuged at 150g for 5 mins. Then the enzyme solution was removed and replaced with serum-free culture media composed of Dulbecco modified Eagle medium (DMEM) and F-12 nutrient (1:1) including insulin (25 µg/mL), transferrin (100 µg/mL), progesterone (20 nM), putrescine (60µM), sodium selenite (30 nM), and human recombinant epidermal growth factor (EGF) 20 ng/mL and fibroblast growth factor-basic (bFGF) 20 ng/mL (R&D Systems, Minneapolis, MN) for 3 days. Upon replating at 1 cell per well in 96-well plates (Corning, Acton, MA), viable cells were counted by trypan blue exclusion (Chiou SH et al, *British J Pharm 2006;48:587-6.*). Then the viable cells were labeled with 1mL CD133/l micromagnetic beads per 1 million cells using the CD133 cell isolation kit (MACS, Miltenyi Biotec).

**The protocol for specific Lentiviral-mediated BCL-2 RNAi**

The siRNA oligonucleotide 5’- AAAATGTGGATGACTGAGTACCTGATTGGAT CCAATCAGGTACTCAGTCATCCACA-3’ targeting human Bcl-2 (NM_000633, nt 1017-1037) was synthesized and cloned into pLVRNAi to generate Leti expression vector, pLVRNAi/Bcl-2. The density of 5 x 10^6^ cells of 293FT cells was plated at per 10 cm plate for one day and then added Lipofectamine 2000 reagent (Invitrongen). The medium with 5ml of Opti-MEM I was replaced without serum. DNA-Lipofectamine 2000 complex with 2 μg of pVSVg, 4 μg of pCMV-ΔR8.91 and 10 μg of pLVRNAi/Oct4 in 3ml of Opti-MEM I were mixed and added into the plate. After 24 hours, the medium containing the complex DNA with culture medium containing serum and antibiotics was replaced. Supernatants were collected 48 hours after transfection and then were filtered; the viral titers were then determined by FACSCalibur apparatus and Cellquest software (BD Biosciences, San Diego,CA) at 48 hours post-transduction. Subconfluent cells were infected with lentivirus at a multiplicity of infection of 5 in the presence of 8 μg/ml polybrene (Sigma-Aldrich).
