# Supplementary material for: Identification of CD133-Positive Radioresistant Cells in Atypical Teratoid/ Rhabdoid Tumor
Source: PLoS One. 2008 May 7;3(5):e2090. doi: 10.1371/journal.pone.0002090 (PMC2396792; doi:10.1371/journal.pone.0002090)
Supplement: Table S1 — (0.06 MB DOC) [file pone.0002090.s005.doc]

**Table S1. Gene name and symbol (Sym)** name

| **Gene Sym** | **Gene Title** | **UGCluster** |
| --- | --- | --- |
| ATR | ataxia telangiectasia and Rad3 related | Hs.271791 |
| BAX | BCL2-associated X protein | Hs.631546 |
| BCL2 | B-cell CLL/lymphoma 2 | Hs.150749 |
| BIRC2 | baculoviral IAP repeat-containing 2 | Hs.643515 |
| CDC25C | cell division cycle 25C | Hs.656 |
| CDK2 | cyclin-dependent kinase 2 | Hs.19192 |
| CHEK2 | CHK2 checkpoint homolog (S. pombe) | HS.291363 |
| CST6 | cystatin E/M | Hs.139389 |
| CYCS | cytochrome c, somatic | Hs.437060 |
| DDX41 | DEAD (Asp-Glu-Ala-Asp) box polypeptide 41 | Hs.484288 |
| MCL1 | myeloid cell leukemia sequence 1 (BCL2-related) | Hs.632486 |
| MDM2 | Mdm2, transformed 3T3 cell double minute 2, p53 binding protein (mouse) | Hs.567303 |
| PAK1 | p21/Cdc42/Rac1-activated kinase 1 (STE20 homolog, yeast) | Hs.435714 |
| PROM1 | prominin 1 | Hs.479220 |
| RAD17 | RAD17 homolog (S. pombe) | Hs.16184 |
| RAD23A | RAD23 homolog A (S. cerevisiae) | Hs.440960 |
| RHOC | Ras homolog gene family, member C | Hs.502659 |
| TP53 | tumor protein p53 (Li-Fraumeni syndrome) | Hs.408312 |
| TP53BP1 | tumor protein p53 binding protein, 1 | Hs.440968 |
| ANTXR1 | Anthrax toxin receptor 1 | Hs.165859 |
| ATM | ataxia telangiectasia mutated (includes complementation groups A, C and D) | Hs.435561 |
| BAK1 | BCL2-antagonist/killer 1 | Hs.485139 |
| BCL2L1 | BCL2-like 1 | Hs.516966 |
| CHEK1 | CHK1 checkpoint homolog (S. pombe) | Hs.24529 |
| H2AFX | H2A histone family, member X | Hs.477879 |
| IL2RA | interleukin 2 receptor, alpha | Hs.467891 |
| LOC440361 | similar to Ig heavy chain V-III region VH26 precursor | Hs.512293 |
| MKI67 | antigen identified by monoclonal antibody Ki-67 | Hs.80976 |
| MMAB | methylmalonic aciduria (cobalamin deficiency) cblB type | Hs.12106 |
| MYC | v-myc myelocytomatosis viral oncogene homolog (avian) | Hs.202453 |
| NBN | nibrin | Hs.492208 |
| PRSS3 | protease, serine, 3 (mesotrypsin) | Hs.128013 |
| RET | ret proto-oncogene (multiple endocrine neoplasia and medullary thyroid carcinoma 1, Hirschsprung disease) | Hs.350321 |
| SERPINA2 | serpin peptidase inhibitor, clade A (alpha-1 antiproteinase, antitrypsin), member 2 | Not found |
| TG | thyroglobulin | Hs.584811 |
| THBD | thrombomodulin | Hs.2030 |
| TNF | tumor necrosis factor (TNF superfamily, member 2) | Hs.241570 |
