# Supplementary material for: Identification of CD133-Positive Radioresistant Cells in Atypical Teratoid/ Rhabdoid Tumor
Source: PLoS One. 2008 May 7;3(5):e2090. doi: 10.1371/journal.pone.0002090 (PMC2396792; doi:10.1371/journal.pone.0002090)
Supplement: Table S2 — (0.04 MB DOC) [file pone.0002090.s006.doc]

**Table S2.** The primers for quantitative RT-PCR

| **Primer** | **Accession No.** | **Sequence** | **Product (bp)** | **Tm (℃)** |
| --- | --- | --- | --- | --- |
| BCL-XL | Z23115 | Sense: CAGGGACAGCATATCAGAG  Antisense: TGGTCATTCAGGTAAGTGG | 184 | 55 |
| BAX | NM_004324 | Sense: GATGCGTCCACCAAGAAG (226)  Antisense: AGTTGAAGTTGCCGTCAG | 163 | 55 |
| MDM2 | BT007258 | Sense: GTAGTAGTCAATCAGCAGGAATC  Antisense: GAAACCAAATGTGAAGATGAAGG | 140 | 52 |
| CDK2 | NM_001798 | Sense: CCTGGACACTGAGACTGAG  Antisense: GTGAGAGCAGAGGCATCC | 183 | 53 |
| CDKN1A | NM_000389 | Sense: TCTACATCTTCTGCCTTAGTCTC  Antisense: TCTTAGGAACCTCTCATTCAACC | 164 | 54 |
| TP53 | NM_000546 | Sense: TGCGTGTGGAGTATTTGGATG  Antisense: GTGTGATGATGGTGAGGATGG | 168 | 55 |
| TP53BP1 | BC112161 | Sense: ATACTTCAGGCAATACTACACATTC  Antisense: TTAGCATCCACATCAGACAGC | 193 | 55 |
| BCL-2 | NM_000657 | Sense: GCGACTCCTGATTCATTGG  Antisense: GTCTACTTCCTCTGTGATGTTG | 162 | 52 |
| CDC25a | NM_201567 | Sense: AAGCGTGTCATTGTTGTG  Antisense: CAGGGTAGTGGAGTTTGG | 118 | 53 |
